# Inherited Heterogeneities Can Control Viscous Subduction Zone Deformation of Carbonates at Seismogenic Depths

**DOI:** 10.1029/2022GL099358

**Published:** 2022-10-07

**Authors:** H. Leah, Å. Fagereng

**Affiliations:** ^1^ Cardiff School of Earth and Ocean Sciences Cardiff University Cardiff UK

**Keywords:** subduction, carbonate, calcite, viscous, creep, heterogeneity

## Abstract

This work links mineral‐scale deformation mechanisms with structural evolution during subduction, providing examples showing how grain‐scale heterogeneities facilitated viscous creep in calcite at nominally seismogenic temperatures. Carbonates commonly enter subduction zones, either highly concentrated in irregularly distributed sediments or as more distributed precipitates in seafloor volcanics. We present shear zones, localized in calcite veins formed during shallow subduction of calcareous sediment and seafloor volcanics, with viscous shear strains of ≥5. Shear strain localized because secondary phases and chemical variations maintained fine grain sizes in calcite aggregates, activating relatively rapid grain size‐sensitive and frictional‐viscous creep at temperatures (260 ± 10°C), cooler than predicted from extrapolation of experimental data. Creep at increased strain rates may limit elastic strain accumulation during interseismic periods, reducing the likelihood of large megathrust earthquakes. As shown here for calcite, common inherited natural heterogeneities may induce weakening of viscous mechanisms in other rocks, or at larger scales in the lithosphere.

## Key Points

- Carbonate sediments and veins are common in subduction zone input sequences and are typically underthrust near the plate interface
- Underthrust carbonates deform faster by dynamic recrystallization and dissolution‐precipitation processes at lower temperature than quartz
- Grain‐scale heterogeneities localize shear strain in calcite veins by promoting viscous deformation at seismogenic zone temperatures

## 1 Introduction

**Figure 1:**
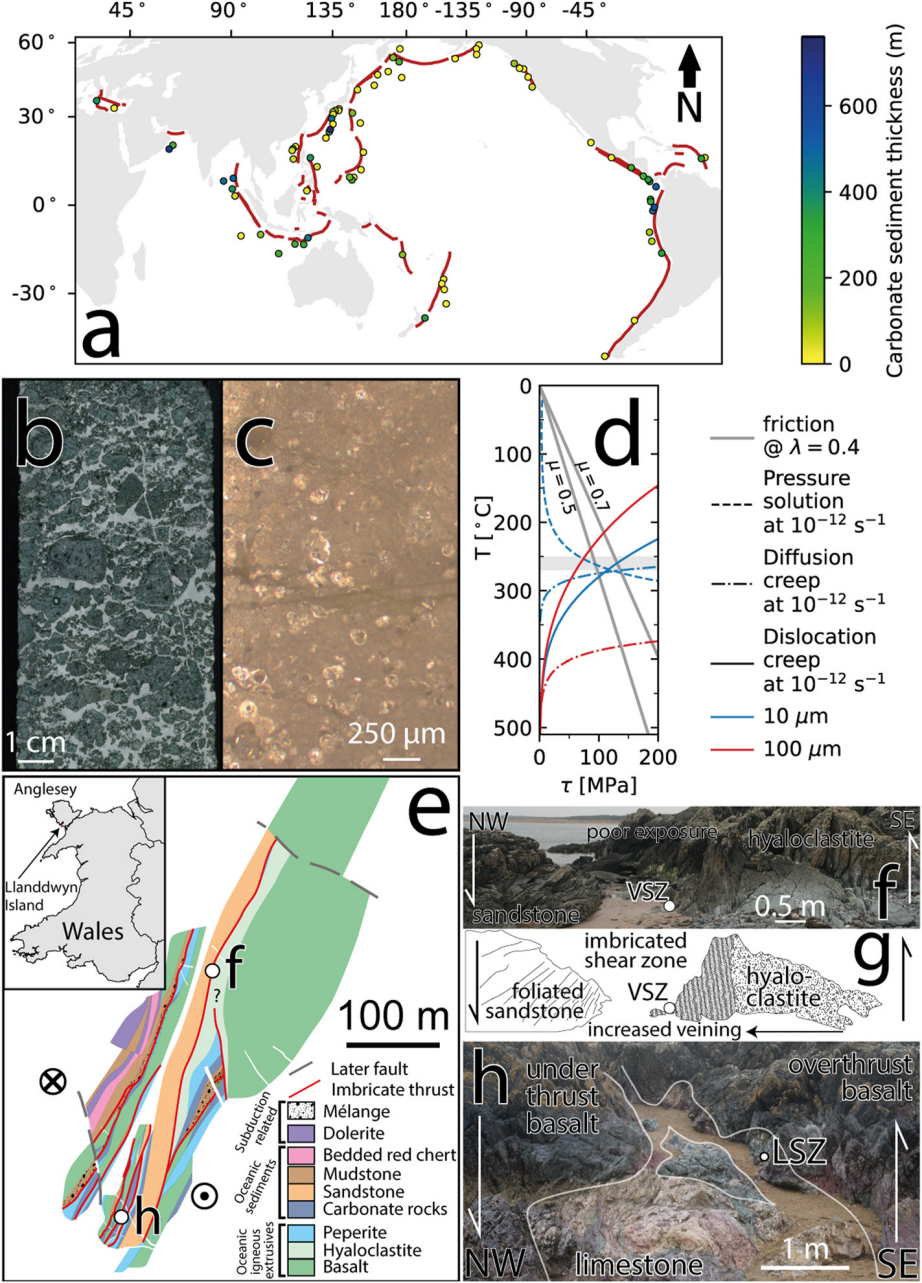
Carbonates in subduction zones. (a) Map of subduction zone ocean drilling sites (IODP, ODP, and DSDP) seaward of subduction zone trenches colored by cumulative thickness of sediment with estimated >30% CaCO_3_ (Table S1 in Supporting Information S1). Core image scans of carbonate‐bearing volcaniclastic lithologies (b) and calcareous pelagic sediments (c) sampled by IODP Exp. 375 (Wallace et al., 2019) and inferred to host the Hikurangi megathrust at seismogenic depths (Barnes et al., 2020). (d) The effect of temperature on the estimated shear stress to deform calcite by pressure solution (Bos, 2002), diffusion creep (Herwegh et al., 2003), and dislocation creep (Renner et al., 2002) at a shear strain rate of 10^−12^ s^−1^ (converted using $\dot{\gamma }=\dot{\varepsilon }\sqrt{3}$). Friction values from Verberne et al. (2015). Gray fill is estimated temperature range of the samples described here (Leah et al., 2022). (e) Sample locations and structure of Llanddwyn Island, Anglesey, UK (adapted from Leah et al., 2022). (f) Field photo and associated sketch (g) showing shear zone hosting the volcanic shear zone sample. (h) Field photo showing shear zone hosting the limestone shear zone sample.

Aseismic creep in subduction zones is common at temperatures <300°C (Bürgmann, 2018; Wallace, 2020), though this is cooler than the base of the subduction megathrust seismogenic zone in many conceptual models (Hyndman & Wang, 1993; Scholz, 1998; Sibson, 1986). Although commonly considered, quartz may not control subduction thrust rheology. The role of phyllosilicates has frequently been considered to explain creep on the subduction thrust (Fagereng & Den Hartog, 2017; French & Condit, 2019); however, carbonates are also a common component of lithological inputs at many subduction margins, and in exhumed accretionary wedges (e.g., Dielforder et al., 2016; Ebert, Herwegh, Evans, et al., 2007; Gray et al., 2007), although the mass of carbonate in actively down‐going plates vary greatly (Figure 1; Table S1 in Supporting Information S1; Plank & Manning, 2019). The majority of carbonate enters subduction zones as sediments (e.g., Figure 1c), though these have the greatest thicknesses at low latitudes and are commonly irregularly distributed along single margins (Figure 1a). Carbonates present within veins throughout volcanic basement (e.g., Figure 1b) are a volumetrically less important but more uniformly distributed source of carbonates to subduction zones (Plank & Manning, 2019). Though many carbonate minerals with distinct rheology occur in the inputs to subduction zones, calcite dominates volumetrically (Underwood, 2007).

Calcite deformation defines a different rheology to silicates with onset of dynamic recrystallization to considerable shear strains at ∼220°C (Austin & Evans, 2007; Ebert, Herwegh, & Pfiffner, 2007; Herwegh et al., 2005, 2011), cooler than the onset of efficient plasticity of quartz at ∼300–350°C (Hirth et al., 2001; Lu & Jiang, 2019; Stipp et al., 2002). To show how aseismic deformation of carbonates can occur at strain rates relevant to actively deforming zones (≥10^−14^ s^−1^; Fagereng & Biggs, 2019), we present examples showing how grain‐scale heterogeneities in subducted carbonates control active deformation mechanisms and strain localization at temperatures of 250–270°C. This work links micro‐scale slip mechanisms to the ongoing evolution of rock volumes being fed into subduction zones and provides mechanistic examples of how viscous creep is facilitated by grain‐scale heterogeneities at typically seismogenic temperatures.

## 2 Carbonate Deformation Mechanisms

Dominant mineral‐scale deformation mechanisms in calcite vary with stress, grain size, and temperature (Figure 1d). Calcite is highly soluble at *T* < 150°C (Plummer & Busenberg, 1982), meaning dissolution‐precipitation creep is typically the dominant calcite deformation mechanism at low pressure and temperatures (Bos, 2002; Zhang et al., 2010). This mechanism leads to a microstructure involving sites of dissolution and nucleation of new grains. Comminution during frictional sliding can also reduce grain size (Sammis & Ben‐Zion, 2008), further increasing the efficiency of grain size sensitive mechanisms including diffusion creep (De Paola et al., 2015; Demurtas et al., 2019; Herwegh et al., 2005). At high temperatures (*T* > 350°C), deformation is dominated by dislocation creep at high stresses and large grain sizes (Renner et al., 2002). Dislocation creep is associated with dynamic recrystallization and recovery, recognized in microstructures such as subgrains and bulging grain boundaries. Low stresses and fine grain sizes lead to dominantly grain size‐sensitive diffusion creep (Figure 1d; Herwegh et al., 2003), associated with nucleation of new grains and growth of existing ones.

Frictional deformation of calcite requires increasing stresses with depth, as effective normal stress increases (Figure 1d). Around temperatures near the top of the seismogenic zone, strain rates from dissolution‐precipitation creep wane (Leah et al., 2020) and stresses required for frictional sliding on calcite become similar to those required to achieve strain rates relevant to deforming zones by dislocation or diffusion creep (Herwegh et al., 2003; Renner et al., 2002). Episodic elevated fluid pressure pulses may cause brief episodes of brittle failure and cataclasis deeper on the megathrust, reducing grain size (Sammis & Ben‐Zion, 2008) and activating grain size‐sensitive mechanisms (De Paola et al., 2015; Demurtas et al., 2019). Viscous mechanisms (i.e., thermally activated, strain‐rate dependent), such as dislocation and diffusion creep, weaken (require lower stresses to achieve the same strain rate) with increasing temperature, becoming increasingly dominant with depth. Viscous deformation of calcite at subduction seismogenic zone temperatures (150 < *T* < 350°C; Hyndman et al., 1997) is commonly attributed to an interplay of dynamic recrystallization by a condition‐dependent combination of grain boundary migration and subgrain rotation, and grain growth by diffusion creep (Bestmann & Prior, 2003; De Bresser et al., 2002). These processes are thought to cause grain size and strain rate convergence around the boundary between dislocation and diffusion creep, sometimes called the field boundary (De Bresser et al., 2001; Etheridge & Wilkie, 1979), but convergence requires both mechanisms to be active. Deformation experiments suggest this likely occurs at temperatures of ≥300°C or ≥20 km depth on subduction plate boundary interfaces (Herwegh et al., 2003; Renner et al., 2002; Syracuse et al., 2010). The seismogenic zone temperature range therefore hosts competition between frictional and viscous mechanisms (Figure 1d), and it is unclear which mechanisms dominate along active, natural subduction megathrusts; this work will address this knowledge gap.

## 3 Geological Setting and Methods

The Gwna subduction complex at Llanddwyn Island (Anglesey, Wales, UK) contains lenticular slices of ocean plate stratigraphy (OPS) metamorphosed to subgreenschist conditions during subduction between 488 and 448 Ma (Kawai et al., 2007). Lenticular slices of OPS are bound on each side by vertical mélange‐bearing shear zones <15 m wide with a SE‐upward shear sense (Figure 1e), defining an imbricated structure consistent in lithology, metamorphic grade, and kinematics with SE‐ward subduction and subsequent passive block rotation of the complex into a vertical dip within regional strike‐slip zones (Leah et al., 2022; Schofield et al., 2021). Massive carbonates and volcanics are commonly deformed alongside varied lithologies in shear zones throughout the complex. Two examples of mm‐scale carbonate shear zones are presented here to highlight how deformation might occur in carbonate sediments (Figure 1h) and carbonate vein‐bearing volcanics (Figures 1f and 1g), referred to as the limestone shear zone and volcaniclastic shear zone, respectively.

Both samples were investigated using optical microscopy, backscattered electron imaging, energy dispersive spectroscopy (EDS), and electron backscatter diffraction (EBSD). Analysis was carried out at the School of Earth and Environmental Sciences at Cardiff University with a Zeiss Sigma HD Field Emission Gun Analytical scanning electron microscope (SEM) fitted with two Oxford Instruments 150 mm^2^ energy dispersive X‐ray spectrometers and a Nordlys EBSD system with Oxford Instruments Aztec software. EDS mapping was performed at 15 or 20 keV accelerating voltage, a beam current of 4.3 nA, aperture of 120 μm, working distance of 8.9 mm, and stepsizes of 1 μm in the limestone and 2 μm in the volcaniclastic shear zone. EBSD was carried out on colloidal silica‐polished thin sections tilted at 70° to the electron beam at a stepsize of 1 μm in the limestone shear zone and 0.7 μm in the volcaniclastic shear zone, working distance of 13 μm, accelerating voltage of 20 keV, beam current of 8.5 nA, and 120 μm aperture. EBSD backscatter patterns were processed using Oxford Instruments Aztec software with a gain of 5 and 2 × 2 binning. EBSD data were then processed using the MTEX toolbox for Matlab (Bachmann et al., 2010) and the methodology of Cross et al. (2017). Phyllosilicate grains were isolated in ImageJ (Schindelin et al., 2012; Schneider et al., 2012) using Mg k*α* EDS map data exported from Oxford Instruments Aztec software. The data were cropped to the vein area and a threshold was applied using the algorithm of Li and Lee (1993) and Li and Tam (1998), resulting in a minimum value of 132 (from a 0–1,778 count range, values below 132 are considered background). Grain shapes, areas, and best fit ellipse dimensions and orientations were then measured in ImageJ. Grain size (d) was calculated as the diameter of a circle with the equivalent area to the grain, $d=2\times \sqrt{\frac{Area}{\pi }}$.

## 4 Carbonate Deformation Microstructures in the Frictional‐Viscous Transitional Temperature Range

### 4.1 Deformation Microstructures in the Limestone Shear Zone

**Figure 2:**
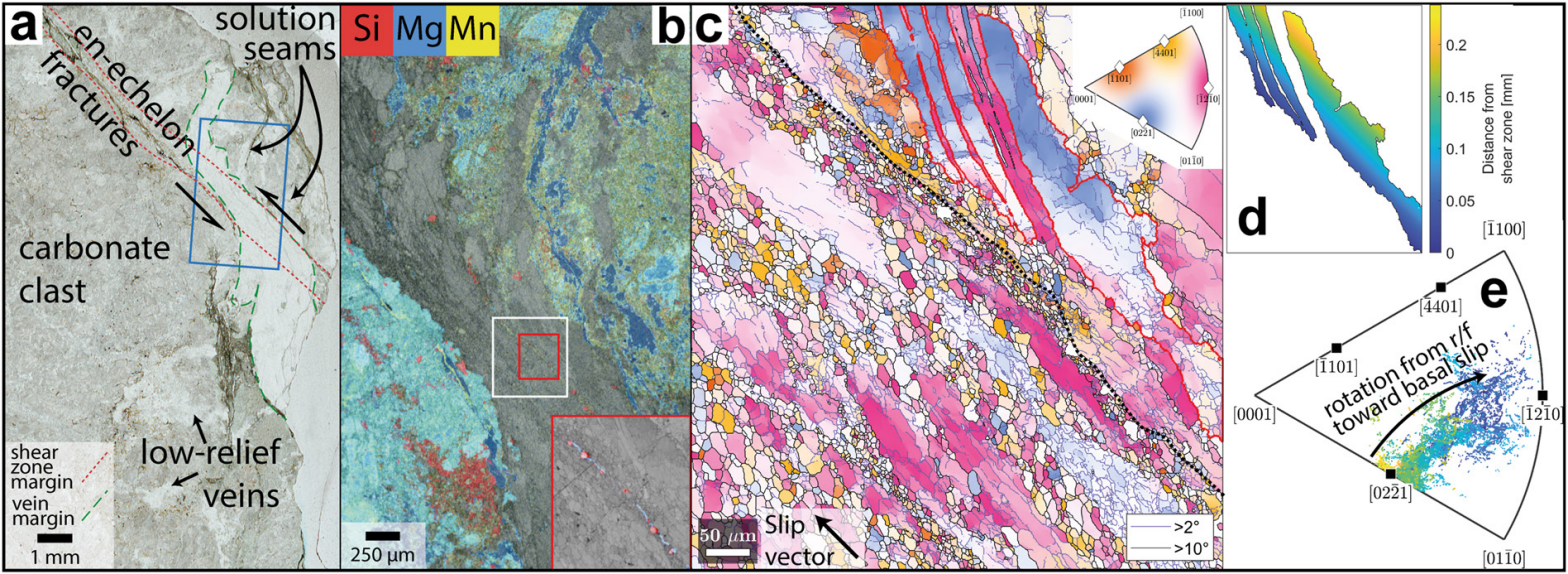
Limestone shear zone microstructure. (a) Optical photomicrograph showing limestone shear zone. Location of (b) is shown as blue rectangle. (b) Scanning electron microscope‐energy dispersive spectroscopy map of shear zone and neighboring clast overlain on an electron backscatter diffraction (EBSD) band contrast image. Inset (red outline) shows detail of quartz (red) and phyllosilicate (blue) grains. Location of (c) is shown as white rectangle. (c) EBSD map colored by crystallographic orientation along the slip vector (as defined by the black arrow), black boundaries are misorientations >10°, purple boundaries are misorientations >2°, red grain boundary outlines grain shown in (d & e), thick black line indicates approximate shear zone boundary. Inset shows key. (d) Large grain highlighted in (c), colored by distance from shear zone boundary. (e) Inverse pole figure of pixel crystallographic orientations along the slip vector, colored by distance from shear zone boundary and within the grain shown in (d).

In the SW of the study area (Figure 1e; Leah et al., 2022) a shear zone with a block‐in‐matrix texture comprises lenses of massive carbonate up to 50 cm long and 30 cm wide in a chlorite matrix (Figure 1h). No clear macroscopic evidence for a shear zone exists but in thin section, massive carbonate clasts dominantly comprise mottled greyish, high optical relief calcite (Figure 2a). The shear zone considered here dips ∼60° SE (∼30° from bulk foliation) and is partially localized within a low optical relief calcite vein. En‐echelon extensional fractures subparallel to the shear zone continue updip from the vein. Outside the shear zone, the calcite vein is offset by ∼400 μm with a reverse sense across a 90 ± 10 μm wide shear zone, consistent with shear strains of 4–5 (Figure 2a).

EDS maps show calcite outside the shear zone contains elevated Mg and Mn concentrations (Figure 2b). Within the shear zone, continuous horizons of equant 5–15 μm grains, including along a through‐going quartz‐chlorite horizon (Figures 2b and 2c), separate grains 30–300 μm across, with aspect ratios ≤8. 30–300 μm grains are elongate parallel to the apparent slip vector (estimated from the 2D shear zone geometry) and some orientations and shapes of some elongate grains match across horizons of finer grains, suggesting they were overgrown by 5–15 μm grains (Figure 2c). 5–15 μm grains are equant and have relatively straight grain boundaries. Subgrains of similar size and shape are common within larger elongate grains (Figure 2c).

EBSD mapping shows scattered average grain orientations outside the shear zone (Figure 2c). Toward the margins of the shear zone, orientations within several grains progressively rotate to align basal *c* slip in the < *a* > direction with the slip vector (Figure 2e). Within the shear zone basal *c* slip in the < *a* > direction is most aligned with the slip vector.

### 4.2 Deformation Microstructures in the Volcaniclastic Shear Zone

**Figure 3:**
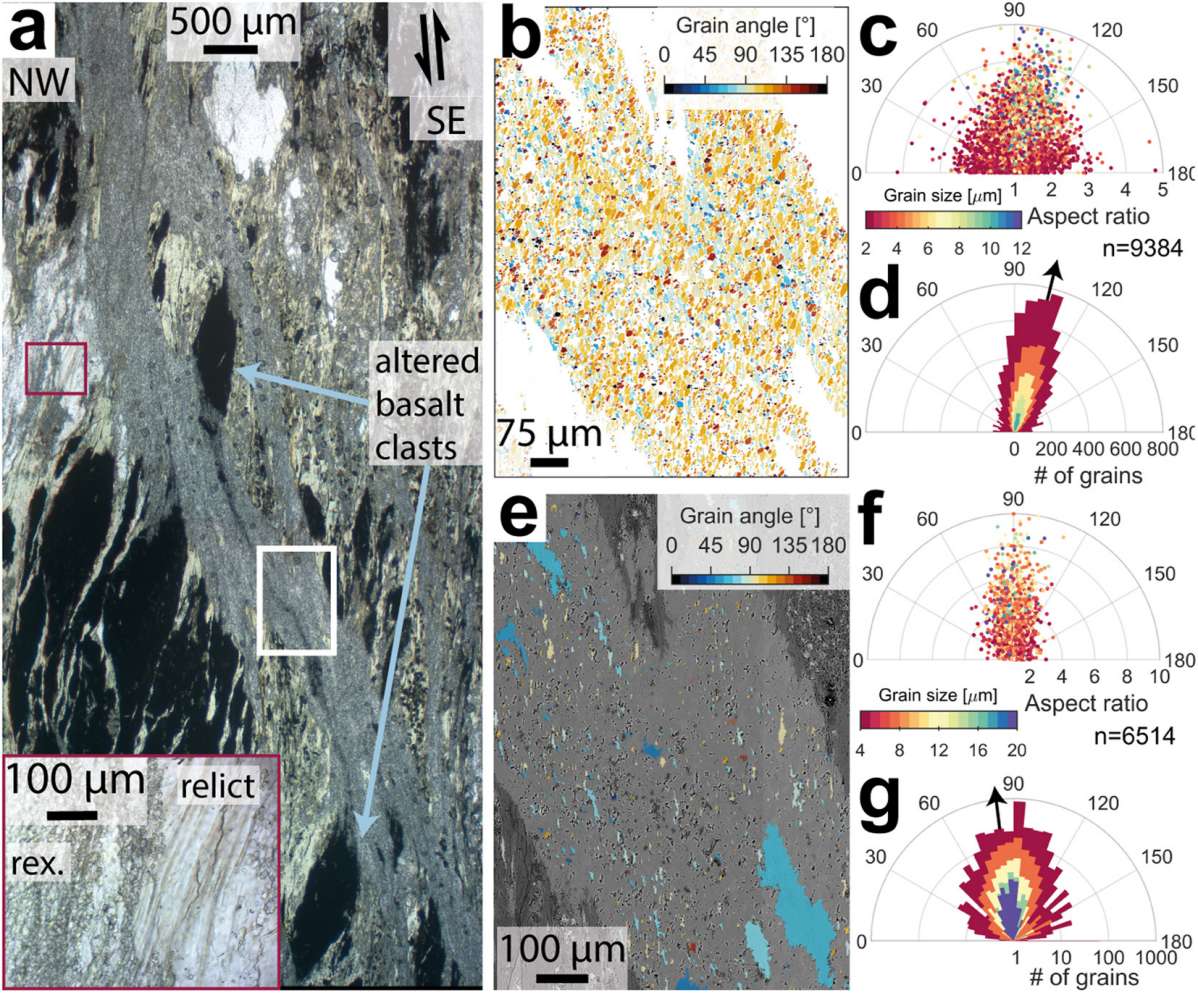
Volcanic shear zone microstructure. (a) Optical photomicrograph of the shear zone, offset altered basalt clasts show shear strains of ∼5. Inset (red outline) shows recrystallized (rex.) grains bordering relict grains where veins aren't through‐going. Location of (b) shown as white rectangle. (b) Calcite grain long axis angle clockwise from horizontal. (c) Calcite grain aspect ratio versus long axis angle. (d) Calcite grain count versus long axis angle. (e) Chlorite grain long axis angle clockwise from horizontal. (f) Chlorite grain aspect ratio versus long axis angle. (g) Chlorite grain count versus long axis angle. Grain angle histograms (d & g) are colored by the minimum grain sizes included in the fraction from their respective color bars, the average orientation of the coarsest fraction is shown as a black arrow.

A shear zone in the north of the study area (Figures 1e–1g; Leah et al., 2022) contains steeply SE‐dipping foliation‐parallel shear zones localized in veins containing fine‐grained dynamically recrystallized calcite (Figure 3a); relict calcite is present outside shear zones. Vein‐localized shear zones cross‐cut the chlorite‐rich matrix and offset altered basalt clasts with a reverse shear sense (SE‐up). <20 μm grains of albite, clay, and chlorite from altered basalt clasts are smeared throughout calcite shear zones, especially where clast fragments are offset across shear zones (Figure 3a). The shear zone has been deformed to shear strains of ≥5 (∼3 mm offsets across a 600 μm wide shear zone; Figure 3a), and comprise minor quartz, albite, clay, and chlorite between <20 μm recrystallized calcite.

Within the shear zone, calcite grains bordering albite and chlorite are <20 μm and have aspect ratios <2.5, whereas calcite grains between these horizons have long axes >20 μm and higher aspect ratios (Figure 3c). The long axes of grains >20 μm plunge steeply toward the NW (Figure 3d), at a low angle to both the shear zone boundary (∼25°), and the bulk chlorite foliation outside the vein (5–15°). Grains <20 μm have a wide distribution of orientations, though most commonly plunge steeply NW, subparallel to grains >20 μm (Figure 3d).

Chlorite grains within the shear zone are mostly <7 μm, with aspect ratios ≤2. Chlorite grains >10 μm in size have greater aspect ratios, reaching >6 (Figure 3f). Chlorite grains <7 μm have a wider distribution of best‐fit ellipse long axis orientations, ±75° from foliation orientation (Figures 3f and 3g). Grains >20 μm are almost exclusively sub‐vertical, ≤30° from the bulk foliation (Figures 3e and 3f). Chlorite grains with aspect ratios >2 occur throughout most of the vein in orientations consistent with the strain field apparent from offset clast fragments (Figures 3a and 3e). Adjacent to calcite grains >20 μm, chlorite grains are generally ≤10 μm and have aspect ratios ≤2 (Figures 3b and 3e).

## 5 Grain‐Scale Mechanisms of Viscous Calcite Deformation

### 5.1 Deformation Mechanisms in the Limestone Shear Zone

In the limestone shear zone, crystallographic orientations in >20 μm relict grains commonly align < *a* > axes with the slip vector (Figures 2c and 2e), suggesting intracrystalline slip occurred by basal glide there. Rotation of crystallographic orientations at the shear zone margin toward those consistent with basal glide shows intracrystalline deformation is spatially associated with the shear zone (Figure 2e). Both Bauer et al. (2018) and Ebert, Herwegh, and Pfiffner (2007) described microstructures consistent with intracrystalline deformation of calcite by basal glide at ≤200°C, possibly assisted by dissolution‐precipitation processes and recovery by cross‐slip. Together with that presented here (Figure 2), these data suggest basal glide is not solely a high temperature calcite deformation mechanism (*T* ≥ 450°C; De Bresser & Spiers, 1997).

Calcite grains >20 μm within the limestone shear zone also host abundant low‐angle grain boundaries (Figure 2c), consistent with dislocation migration enabling subgrain formation and subsequent rotation recrystallization (Rutter, 1995). Mg and Mn‐rich regions outside the shear zone host little apparent strain (Figure 2b). Experiments show Mn enhances strain rate of grain boundary diffusion and that Mg content increases the strength of carbonates undergoing dislocation creep (Xu et al., 2009). We suggest the coarseness of grains outside the shear zone limited the weakening effect of Mn, instead higher Mg content inhibited efficient dislocation migration and dynamic recrystallization. Dislocation migration in the low‐Mg shear zone led to dynamic recrystallization, grain size reduction, and subsequent grain size‐sensitive creep. Nucleation and growth of secondary chlorite possibly consumed Mg and Mn from shear zone calcite by diffusion, enhancing their depletion by deformation (Herwegh et al., 2011). We cannot decipher whether these compositional variations are primary or secondary, but emphasize that variation itself promotes strain localization.

### 5.2 Deformation Mechanisms in the Volcaniclastic Shear Zone

No evidence of intracrystalline deformation, such as aligned crystallographic orientations or elongate coarse grains, is observed in the volcaniclastic shear zone (Figure S1 in Supporting Information S1). Possibly because secondary chlorite throughout the vein (Figure 3e) inhibited growth of coarse calcite grains, in which higher strain rate dislocation creep occurs (Renner et al., 2002). Deformation of similar phyllosilicate‐calcite aggregates from the Doldenhorn nappe was interpreted to occur by grain boundary sliding and stress‐driven solution transfer in fine‐grained areas, with intracrystalline plasticity in coarser areas (Herwegh & Berger, 2004; Herwegh & Jenni, 2001). The volcaniclastic shear zone deformed at lower temperatures than the aggregates described by Herwegh and Berger (2004), limiting intracrystalline deformation and making diffusive mass transfer the dominant mechanism in areas with grains >20 μm, as also reported at ∼200°C on the Glarus thrust by Ebert, Herwegh, and Pfiffner (2007). This interpretation is consistent with elongate grains with long axes >20 μm normal to the interpreted principal stress direction (Figure 3b; Rutter, 1976).

Chlorite in the volcaniclastic shear zone likely nucleated into voids formed by grain boundary sliding (Paterson, 1995) before rotating and growing by solid solution (*sensu* Herwegh & Jenni, 2001). Solute likely originated locally from chlorite grain boundaries oriented oblique to the principal stress direction (Rutter, 1976), increasing chlorite aspect ratios (Figures 3a and 3e) and potentially providing a sink for Mg and Mn from strained calcite. Chlorite grains >10 μm are spatially associated with <20 μm calcite grains (Figures 3b and 3e) suggesting a combination of grain‐parallel slip (*sensu* Okamoto et al., 2019) in coarsening chlorite grains, and grain boundary sliding of dynamically recrystallizes calcite grains occurred there. >20 μm calcite grains occur in areas containing smaller chlorite grains with lower aspect ratios and widely distributed orientations (Figures 3b–3e). We interpret these areas as lower shear strain because larger calcite grains have more difficulty sliding past one another.

### 5.3 The Importance of Heterogeneities

Grain boundary sliding coupled to dissolution‐precipitation processes is more efficient at finer grain sizes (Herwegh et al., 2011; Rutter, 1976), so grain size reduction by dynamic recrystallization has often been associated with rheological weakening, though it is likely a competition between grain size reduction by subgrain rotation recrystallization and grain growth by grain boundary migration (De Bresser et al., 2001). Both shear zones presented here have secondary phases that pin grain boundaries, inhibiting growth (Figures 2b and 3e). If coarse grains are necessary for dislocation creep in calcite, as the Hall‐Petch relation (Renner et al., 2002) and field‐boundary hypothesis (De Bresser et al., 2001) imply, distributed secondary phases throughout the volcanic‐hosted calcite vein may inhibit dislocation creep by limiting grain growth, as invoked in the retrograde Glarus thrust at similar (but decreasing rather than increasing) temperatures (Ebert, Herwegh, Evans, et al., 2007; Ebert, Herwegh, & Pfiffner, 2007). Alternatively, the vein may deform by sliding on grain boundaries and aligned chlorite grains, coupled to dissolution‐precipitation creep, at stresses below those required to activate dislocation creep (Herwegh & Jenni, 2001; Okamoto et al., 2019). Either way, deformation and rheology within the volcaniclastic shear zone was limited to mechanisms enabled by secondary included phases. In contrast, deformation of the limestone shear zone was more strongly controlled by the evolution of calcite grain size (Figure 2b). Shear offsets are larger across finer‐grained horizons, consistent with rheological weakening due to secondary phases pinning grain boundaries (Figure 2c). Away from secondary phases, grain sizes are larger and the competition between diffusion creep accompanied by grain growth and dynamic recrystallization resulting in grain size sensitive flow is more apparent (Figure 2c).

## 6 The Role of Inheritance in Subduction Deformation of Carbonates

Both shear zones presented here localized within calcite‐filled veins that likely formed by brittle deformation near the megathrust toe (Figures 2a and 3a), where effective normal stresses are lower (Sibson, 1998) and low temperatures mean calcite solubility is high (Plummer & Busenberg, 1982). As temperatures increase with increasing depth, calcite solubility and associated solute concentration reduce until negligible around the updip end of the seismogenic zone (Leah et al., 2020). The onset of dynamic recrystallization in calcite has been estimated to occur around 200°C (Ebert, Herwegh, & Pfiffner, 2007; Herwegh et al., 2005). The dominant deformation mechanism in calcite also depends on strain rate (Rogowitz et al., 2014), however, such that this temperature varies with regional strain rate and degree of localization. By inducing localization of grain size reduction and/or maintaining finer grain sizes, inherited heterogeneities (e.g., chemical variation, abundant secondary phases) can induce weakening and localization of viscous deformation mechanisms in calcite at seismogenic zone temperatures (260 ± 10°C; Leah et al., 2022). This results from diffusive processes that are more efficient at small grain sizes, and a shallower onset of the frictional‐viscous transition than would be expected from dynamic recrystallization alone (Bauer et al., 2018; Ebert, Herwegh, Evans, et al., 2007).

Most natural rocks likely contain secondary phases and local chemical variations, suggesting grain‐scale heterogeneities may have a more widespread role controlling weakening and strain localization of viscous deformation mechanisms than typically considered. Onset conditions for viscous creep in calcite have important implications for estimated mid‐crustal deformation rates in carbonate terranes, and similar rheological effects of heterogeneities may be important for other minerals (e.g., quartz, olivine; Herwegh et al., 2011) in the crust and mantle.

## 7 The Role of Carbonate in Subduction‐Related Creep, Slow Slip, and Earthquakes

**Figure 4:**
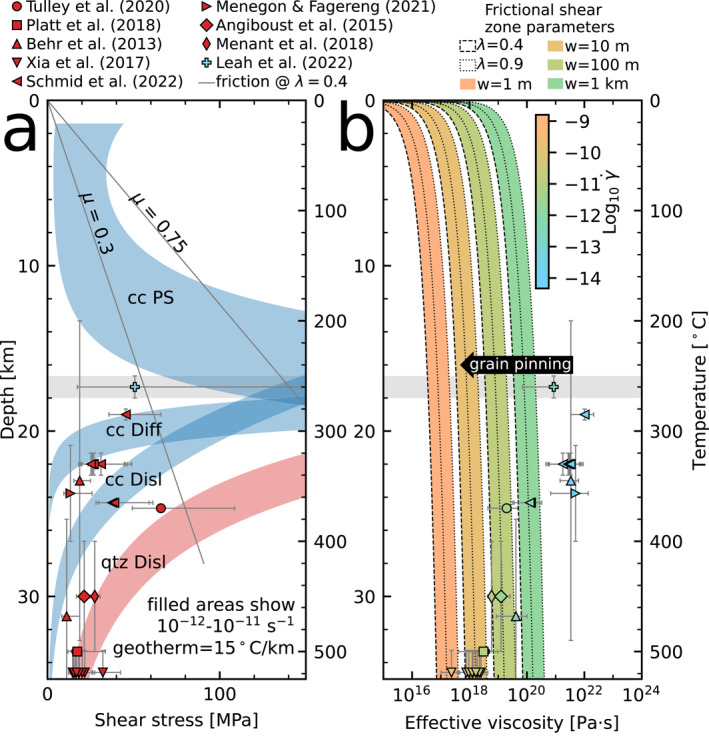
Strength and effective viscosity of carbonates in subduction zones. (a) Strength versus depth/temperature from flow laws for calcite (cc) pressure solution (Bos, 2002), calcite dislocation creep (Renner et al., 2002), calcite diffusion creep (Herwegh et al., 2003), and quartz dislocation creep (Lu & Jiang, 2019). Temperature‐quartz piezometer stress estimates from various sources (red points) are plotted alongside estimates from monomineralic areas of samples presented in this study (Leah et al., 2022). (b) Effective viscosity with depth for strength estimates shown in A, with strain rates for quartz from the flow law of Lu and Jiang (2019) and for calcite from summing Herwegh et al. (2003) and Renner et al. (2002), assuming deformation by diffusion and dislocation creep. Also shown are effective viscosities for frictional sliding for various shear zone widths and pore fluid factors (see legend) assuming a plate velocity of 50 mm yr^−1^. Color scale show Log_10_ shear strain rate and is the same for frictional and viscous mechanisms. Gray fill shows temperature range of deformation described here (Leah et al., 2022), where heterogeneities such as grain pinning can reduce effective viscosity in the frictional‐viscous transition.

As carbonates generally appear in the lowest part of the sedimentary section or top of the oceanic crust (Table S1 in Supporting Information S1; Alt & Teagle, 1999; Gillis & Coogan, 2011), they are likely to be underthrust near the plate interface (Barnes et al., 2020; Meneghini et al., 2009). Long‐term slip rates from frictional or brittle deformation during subduction, at margins subducting calcite content above some threshold value (Figure 1a), are likely overtaken by those from viscous mechanisms (combinations of dislocation and diffusion creep) around the frictional‐viscous transition in calcite (Figure 4; 200–300°C). Strain within shear zones under prograde conditions would therefore progressively localize in volumes deforming by viscous mechanisms, possibly initial precursory geometries such as the calcite veins presented here (*sensu* Mancktelow & Pennacchioni, 2005).

Flow laws predict viscous creep occurs at low strain rates in the frictional‐viscous transition (Figure 4a), requiring km‐wide shear zones to accommodate plate rate convergence (Figure 4b). Microstructures presented here show meaningful strains (4–5) accommodated over mm‐scale shear zones (Figures 2a and 3a), meaning it is likely grain‐scale heterogeneities caused rheological weakening and strain localization into mm‐scale shear zones at strain rates higher than predicted from extrapolation of flow laws (Figure 4a). Heterogeneities (e.g., grain pinning here) appear to have caused relatively rapid creep by viscous mechanisms, reducing the effective viscosity of calcite in shear zones at temperatures consistent with the frictional‐viscous transition, where high stresses and effective viscosity are associated with low strain rates (Figure 4b). High stresses in this temperature range (Figure 4a) are also associated with seismicity, but higher viscous strain rates may suppress this by limiting accumulated elastic strain during interseismic periods.

Recent work has highlighted heterogeneity as an important control on subduction zone deformation style (e.g., Barnes et al., 2020). The role of heterogeneity in localizing viscous deformation highlighted here may also be applicable to larger rock units (e.g., sedimentary beds, crustal volumes). Scaling of heterogeneities is likely controlled by a combination of (a) the relative scales and distribution of heterogeneities and considered volumes, and (b) the effectiveness of a heterogeneity at weakening or strengthening the considered mechanisms. In the case of calcite‐bearing thrusts, upscaling from mm‐scale shear zones documented in detail here to km‐scale behavior is speculative but supported by observations from the exhumed Glarus thrust, in the Helvetic Alps, which accommodated displacements ≤50 km on 0.1 − >10 m thick shear zones, where deformation localized with comparable temperatures and mechanisms to those described here (Ebert, Herwegh, Evans, et al., 2007; Ebert, Herwegh, & Pfiffner, 2007). Whereas Glarus is an out‐of‐sequence thrust where localization is linked to decreasing temperature with increasing strain (Ebert, Herwegh, & Pfiffner, 2007), Dielforder et al. (2016) demonstrated the prograde deformation of accreted sediments, including carbonates, follow similar deformation paths to those inferred for active subduction zones ‐ including progressive veining and deformation of such veins.

If viscous deformation of carbonates, including by competing diffusion and dynamic recrystallization processes from about 200°C, is important in active subduction zones, then there should be margins creeping at this temperature. Although creep in this temperature range may also be accommodated by frictional‐viscous shear of quartz‐phyllosilicate mixtures under some conditions (Bos, 2002; Fagereng & Den Hartog, 2017), we note that carbonates are present and may correlate with creeping patches at least in Costa Rica (Ikari et al., 2013) and northern Hikurangi (Barnes et al., 2020). This correlation, however, remains speculative and future, more detailed work on linking input sediments and down‐dip megathrust patches of different behavior may further test this hypothesis.

## Supporting information

Supporting Information S1

## Data Availability

Data used in this work are publicly available from Leah (2022) (https://doi.org/10.5281/zenodo.6504410).
