# Supplementary material for: Inherited Heterogeneities Can Control Viscous Subduction Zone Deformation of Carbonates at Seismogenic Depths
Source: Geophys Res Lett. 2022 Oct 7;49(19):e2022GL099358. doi: 10.1029/2022GL099358 (PMC9788063; doi:10.1029/2022GL099358)
Supplement: Supplementary file 1 — Supporting Information S1 [file GRL-49-e2022GL099358-s001.pdf]

Supporting Information for "Inherited heterogeneities can control viscous subduction zone deformation of carbonates at seismogenic depths"

H. Leah[1]

Å. Fagereng[1]

[1]Cardiff School of Earth and Ocean Sciences, Cardiff university, Main building, CF10 3AT

Contents of this file

1. Figure S1
2. Captions for Datasets S1 to S3
3. Caption for Table S1

Additional Supporting Information (Files uploaded separately)

1. Datasets S1 to S3
2. Table S1

Introduction

This supplementary information contains a figure showing, and captions for, EBSD, EDS, and BSE data collected on samples from the Gwna subduction complex at Llanddwyn Island, Anglesey, Wales, UK between 2018 and 2020. EBSD data are shared at .ctf

files exported from Aztec. Grains were constructed following the methodology of (Cross et al., 2017). EDS data are shared as .tif files where each pixel value corresponds to counts at that pixel. Table S1 is uploaded separately as a .csv file. Datasets S1-S3 and Table S1 have been deposited in Zenodo at Leah (2022, DOI: 10.5281/zenodo.6504410).

Figure S1. - EBSD map showing crystallographic orientations of slip vector for volcaniclastic vein

Data Set S1. - Clast shear zone EBSD data

EBSD data used to construct Fig. 2c-e, includes band contrast used as the background of Fig. 2b.

Data Set S2. - Volcanic shear zone EBSD data

EBSD data used to construct Fig. 3b-d.

Data Set S3. - Volcanic shear zone chemical and chlorite data

EDS and backscatter electron image used to construct Fig. 3e-g. Includes grain size results from ImageJ.

Table S1. - Carbonate sediments cored by ocean drilling projects

Carbonate sediment thicknesses ($> 40\%$ $CaCO_3$) drilled seaward of subduction zones from IODP, DSDP, and ODP databases. Note that thicknesses do not necessarily correspond to the difference between top and bottom depth due to discontinuous thicknesses calcareous sediments.

References

Cross, A. J., Prior, D. J., Stipp, M., & Kidder, S. (2017, jul). The recrystallized grain size piezometer for quartz: An EBSD-based calibration. *Geophysical Research Letters*, *44*(13), 6667–6674. Retrieved from http://doi.wiley.com/10.1002/2017GL073836 doi: 10.1002/2017GL073836

Leah, H. (2022). *Supplementary information for "Inherited heterogeneities can control viscous subduction zone deformation of carbonates at seismogenic depths".* doi: 10.5281/zenodo.6504410

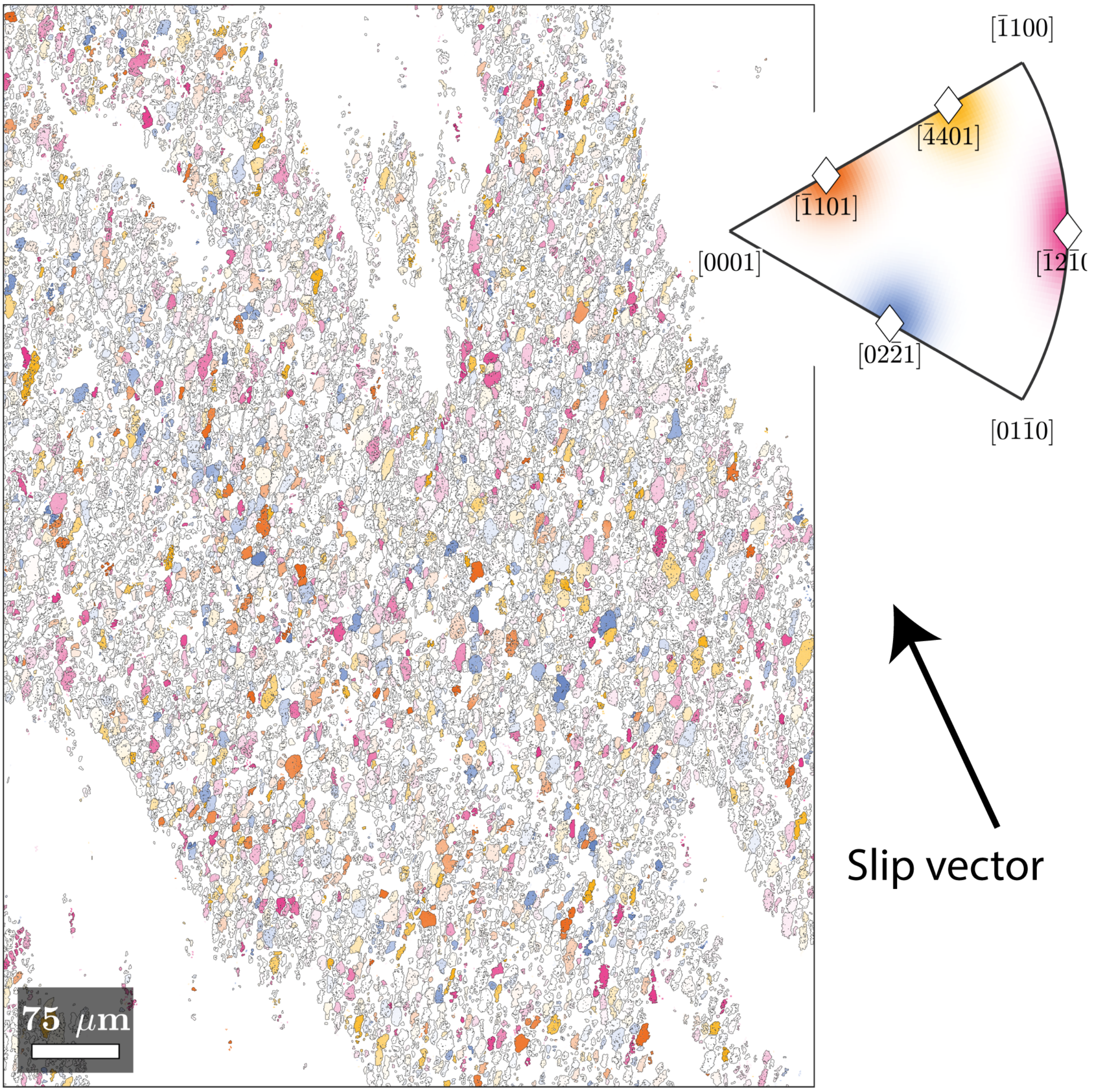

Figure S1. EBSD map of volcanic shear zone (area shown by white rectangle in Fig. 3a) showing crystallographic orientations of the interpreted shear zone slip vector.
